# Non-Surgical Periodontal Therapy with Adjunctive Amoxicillin/Metronidazole or Metronidazole When No *Aggregatibacter actinomycetemcomitans* Is Detected—A Randomized Clinical Trial

**DOI:** 10.3390/antibiotics9100686

**Published:** 2020-10-09

**Authors:** Holger F. R. Jentsch, Martin Dietrich, Sigrun Eick

**Affiliations:** 1 Centre for Periodontology, Department of Cariology, Endodontology and Periodontology, University Hospital of Leipzig, Liebigstr. 12, Haus 1, D-04103 Leipzig, Germany; 2 Private Dental Practice, Borngasse 12, D-99084 Erfurt, Germany; za.m.dietrich@gmail.com; 3 Department of Periodontology, Laboratory of Oral Microbiology, School of Dental Medicine, University of Bern, Freiburgstr. 7, CH-3010 Bern, Switzerland; sigrun.eick@zmk.unibe.ch

**Keywords:** scaling and root planing, antibiotics, periodontitis, non-surgical treatment

## Abstract

Background: The aim was to compare two different systemic antibiotics regimens adjunctive to non-surgical periodontal therapy when *Aggregatibacter actinomycetemcomitans* was not detected in the subgingival biofilm. Methods: A total of 58 patients with periodontitis and with no *A. actinomycetemcomitans* in the subgingival biofilm were treated with full-mouth subgingival instrumentation and either metronidazole (MET; *n* = 29) or amoxicillin/metronidazole (AMX/MET; *n* = 29). Probing depth (PD), clinical attachment level (CAL) and bleeding on probing (BOP) were recorded at baseline, as well as after three and six months. Subgingival biofilm and gingival crevicular fluid were collected and analyzed for major periodontopathogens and biomarkers. Results: PD, CAL and BOP improved at 3 and 6 months (each *p* < 0.001 vs. baseline) with no difference between the groups. Sites with initial PD ≥ 6 mm also improved in both groups after 3 and 6 months (*p* < 0.001) with a higher reduction of PD in the AMX/MET group (*p* < 0.05). T. forsythia was lower in the AMX/MET group after 3 months (*p* < 0.05). MMP-8 and IL-1β were without significant changes and differences between the groups. Conclusion: When *A. actinomycetemcomitans* was not detected in the subgingival biofilm, the adjunctive systemic use of amoxicillin/metronidazole results in better clinical and microbiological outcomes of non-surgical periodontal therapy when the application of systemic antibiotics is scheduled.

## 1. Introduction

Periodontitis is a chronic inflammatory, autodestructive disease [1,2] with high prevalence [3,4,5]. The tooth-supporting tissues are progressively destructed and subsequent tooth loss can occur. Subgingival instrumentation is an efficacious part of the systematic periodontal treatment [6,7,8,9,10]. Systemic antimicrobials as an adjunct to subgingival instrumentation statistically significantly improve the clinical results of the nonsurgical therapy. There exist proves of positive effects of several antibiotics regarding the reduction of probing depth (PD) and bleeding on probing (BOP) and additional attachment gain (CAL) is also reported [11]. The adjunctive use of antibiotics is not without any concern respecting public and patient´s health. Following this, it is not recommended to treat all periodontitis patients routinely with adjunctive antibiotics (Treatment of stage I-III Periodontitis) [12]. However, higher initial probing depths benefit from systemic antibiotics adjunctively given to the subgingival instrumentation [13]. Moreover, the number of sites to be treated surgically is diminished by adjunctive antibiotics to subgingival instrumentation [11].

Several bacterial species, such as *Aggregatibacter actinomycetemcomitans* [14,15], *Porphyromonas gingivalis* [15,16], *Treponema denticola*, and *Tannerella forsythia* [17,18], trigger the local inflammation and are proven to damage and destroy periodontal tissues.

The capnophilic *A. actinomycetemcomitans* is most sensitive to amoxicillin but resistant to metronidazole, while the other Gram-negative but strictly anaerobe bacteria (e.g., *P. gingivalis*) are most highly sensitive to metronidazole [19,20,21]. Contrastingly, there is not always a high susceptibility of bacteria in the subgingival biofilm to amoxicillin [22]. This raised the question as to whether, in the absence of *A. actinomycetemcomitans*, the administration of amoxicillin could be avoided.

The combination of amoxicillin and metronidazole is widely used in the periodontal therapy [23]. Empiric adjunctive use of antibiotics without testing the composition of the subgingival biofilm results mostly in the prescription of the combination of amoxicillin and metronidazole [24]. On the other hand, studies and reviews describing the positive results of the adjunctive medication with amoxicillin and metronidazole did not differentiate between *A. actinomycetemcomitans*-positive or -negative tested biofilms or patients.

To the best of the authors’ knowledge, no study has been performed to compare the clinical results and the effects on several periodontopathogens and biomarkers after administration of amoxicillin and metronidazole or metronidazole alone in *A. actinomycetemcomitans*-negative patients adjunctively prescribed to subgingival instrumentation. The aim of the study is to verify if there exist differences between the prescription of solely metronidazole versus the combined antibiotic therapy with amoxicillin and metronidazole in patients where an adjunctive antibiotic regime is scheduled. The hypothesis of the present study was that the prescription of amoxicillin/metronidazole results in better outcomes at the clinical variables PD, CAL and BOP than the use of solely metronidazole as an adjunct to subgingival instrumentation in patients tested negative for *A. actinomycetemcomitans* in the subgingival biofilm.

## 2. Materials and Methods

### 2.1. Patients

The study protocol was approved by the Ethics Committee at the Medical Faculty, Leipzig University (078-16-14032016) and followed the principles of the Declaration of Helsinki, as revised in 2013. It is deposited at the German register of clinical trials (DRKS00015193). The study participants were recruited and treated in a private dental practice with experiences in the field of periodontology (M.D.). The treatment was supervised by a specialist for periodontology (H.J.). Every volunteer was fully informed and gave written consent to participate in the study.

Criteria for being included in the study were having no less than 16 teeth (minimum four in each quadrant) and at least two independent sites with probing depths ≥ 6 mm per quadrant and a full mouth plaque score below 35% after two sessions of professional prophylaxis and instruction similar to the inclusion criteria of Haffajee et al. (2007). Exclusion criteria were antibiotic treatment within the last three months, periodontal therapy within the last year, pregnancy or breastfeeding period and conditions or systemic diseases affecting the periodontal tissues. A further exclusion criterion was the detection of *A. actinomycetemcomitans* in the subgingival biofilm tested by analysis of the subgingival biofilm two weeks before subgingival instrumentation. From the recruited 82 patients, four refused to participate and 23 had to be excluded due to the presence of *A. actinomycetemcomitans* in the subgingival biofilm or other reasons for exclusion. Following this, 58 participants remained in the study. The allocation to the two study groups was made with a computer-generated table in a ratio 1:1. All examinations and the treatment were performed by one clinician (M.D) who was unaware about the study group. The delivery of the assigned antibiotics was made by a dental assistant of the dental practice (M.W.) who was not involved in the treatment of the patient. Unblinding of the data was made after recording all patients’ data. The prior intra-examiner calibration performed by repeated measurements of PD in two quadrants of eight patients resulted in κ = 0.88.

### 2.2. Therapy and Follow-Up Treatment

Both groups received the same full-mouth subgingival instrumentation in one session within four weeks after baseline examination (after receiving the results of the microbiological testing of the subgingival biofilm for *A. actinomycetemcomitans*). Subgingival instrumentation was conducted by using articaine hydrochloride/epinephrine hypochloride for local anesthesia (Ultracain D-S, Sanofi-Aventis, Frankfurt/Main, Germany) and hand (Gracey Curettes Mini Five rigid, Hu-Friedy Manufacturing Co., Chicago, IL, USA) and ultrasonic instruments (EMS SA, Nyon, Switzerland) for the subgingival instrumentation. The test (metronidazole (MET)) group received 3 × 400 mg metronidazole from the first day of subgingival instrumentation, and the control (amoxicillin (AMX)/MET) group received a combination of 3 × 400 mg metronidazole and 3 × 500 mg amoxicillin both for one week. The drug packages were not blinded. In the first week after treatment, each individual performed normal careful oral hygiene with tooth and interdental brushes supplemented by one minute rinsing with a chlorhexidine digluconate mouthrinse (Chlorhexamed forte 0.2%, GlaxoSmithKline Healthcare, Bühl, Germany) twice a day. Exact drug use was reinforced by telephone calls. For the case of any adverse effect the patients were instructed to address the dental practice. After three and six months, the participants received supportive periodontal treatment including re-motivation and reinstruction.

### 2.3. Clinical Variables and Sampling Procedures

The recording of the clinical data and the collecting of biological materials were made at three appointments, at baseline before subgingival instrumentation (t0) and three (t3) and six months (t6) after subgingival instrumentation. Patients were examined for PD, CAL and BOP and the full-mouth plaque score (FMPS). PD, CAL and BOP were determined in a six-point measurement per tooth (mesiobuccal, distobuccal, buccal, mesiooral, distooral and oral) using a manual periodontal probe (PCVNCKIT6, Hu-Friedy Manufacturing Co., Chicago, IL, USA).

For the collection of the gingival crevicular fluid (GCF), sterile paper strips (Periopaper, Oraflow Inc., Smithtown, NY, USA) were left at the pocket entrance of the deepest pocket at baseline in each quadrant for 30 s. The periopaper strips were transferred into Eppendorf safe-lock tubes, containing proteinase inhibitor (Sigma-Aldrich, St. Louis, MO, USA), and stored for a few days at −20 °C and thereafter at −80 °C until assayed. Samples of the subgingival biofilm were collected at the same sites by inserting a paper point (ISO 60, Roecko, Langenau, Germany) for 30 seconds. All collected GCF and biofilm samples were analyzed at the Laboratory of Oral Microbiology, Department of Periodontology, School of Dental Medicine, University of Bern, Bern, Switzerland.

### 2.4. Biomarkers and Microbiological Analysis

Prior to the analysis, GCF samples were eluted at 4 °C overnight in 750 µL phosphate-buffered saline. From the eluates, the levels of interleukin (IL)-1β and matrix-metalloprotease (MMP)-8 were determined by using commercially available enzyme-linked immunosorbent assay (ELISA) kits (R&D Systems Europe Ltd., Abingdon, UK). These kits had detection levels of 1 pg/site for IL-1β and 100 pg/site for MMP-8 and were used according to the instructions of the manufacturer.

From all subgingival biofilm samples, DNA was extracted by using Chelex method [25]. Samples taken at baseline were screened for presence of *A. actinomycetemcomitans* by using real-time PCR [26]. When being negatively tested for A. actinomycetemcomitans, those samples and all samples collected at the follow-up time points were processed as described recently [27]. In short, a multiplex-real-time qPCR was performed that quantified *A. actinomycetemcomitans*, *P. gingivalis*, *T. forsythia* and *T. denticola* by using specific primers and probes. The results of the bacterial counts are given in log10. The sensitivity for the method is 102 for each bacterial species.

### 2.5. Data Analysis

The clinical and laboratory data were statistically analyzed with the Statistical Package for Social Sciences (SPSS version 24.0 SPSS Inc., Chicago, IL, USA). The primary outcome variable was the change of PD in periodontal pockets with initial PD ≥ 6 mm after six months. Based on the data of Haffajee et al. [28], a mean difference of 0.26 mm in observed PD in initial sites with PD ≥ 6 mm with a standard deviation of 0.32 mm between the two groups dates would require ≥ 24 patients per group in order to detect a significant difference (*p* ≤ 0.05) with a test power of 80%.

Secondary outcome variables were changes of mean PD, BOP, CAL and the number of sites with PD ≥ 6 mm, levels of IL-1β and MMP-8 as well as the CFU of the studied periodontopathogenic bacteria after three and six months. All analyses assumed the patient as the statistical unit. Nonparametric tests (Friedman/Wilcoxon) were used for intra-group testing, and the Mann–Whitney U test was used for inter-group testing. The level of significance was α ≤ 0.05.

### 2.6. Ethics Statements

All procedures performed in studies involving human participants were in accordance with the ethical standards of the institutional and/or national research committee and with the 1964 Helsinki declaration and its later amendments or comparable ethical standards.

### 2.7. Informed Consent

Informed consent was obtained from all individual participants included in the study.

## 3. Results

### 3.1. Study Population and Clinical Variables

**Figure 1:**
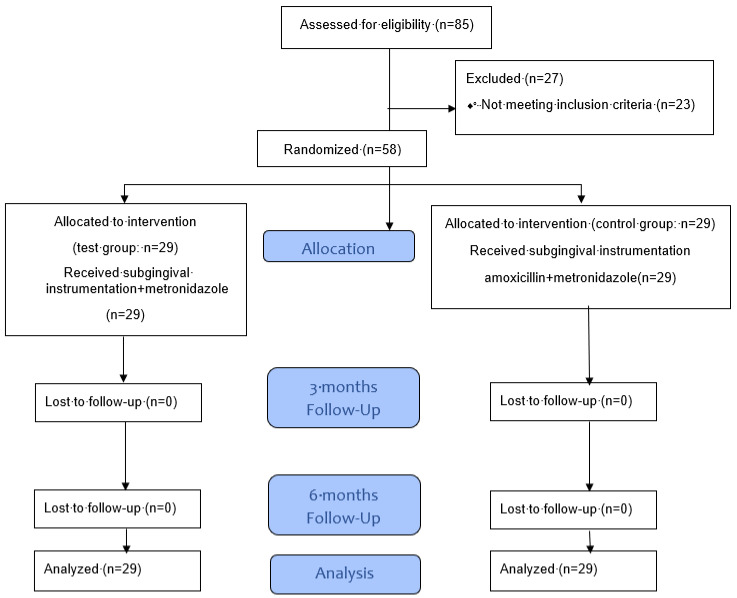
Flowchart (adapted to Moher et al. [29]) of the study non-surgical periodontal therapy with adjunctive amoxicillin/metronidazole or metronidazole in the absence of *Aggregatibacter actinomycetemcomitans*—a randomized clinical trial.

This randomized controlled trial took place during July 2016 and June 2018. From the primary, cohort 28 patients were excluded for not matching the criteria or for refusing to participate. A total of 29 patients per group entered the study. The demographic data are shown in Table 1, and the study flow is given in the flowchart adapted to Moher et al. [29] in Figure 1. The patients had on average 25.7 ± 3.1 teeth and 15.1 ± 5.7 teeth with PD ≥ 6 mm in the AMX/MET and 25.7 ± 3.1 teeth and 14.7 ± 7.8 teeth with PD ≥ 6 mm in the MET group (*p* = 0.561; *p* = 0.488). None of the individuals reported any adverse effects from the prescribed antibiotics or the subgingival instrumentation.

The clinical results are given in Table 2. In both groups, the subgingival instrumentation with adjunctive antibiotic therapy resulted in significant improvements of PD, CAL and BOP after three and six months (all *p* < 0.001). The mean PD of the sites with initial PD ≥ 6 mm as well as the number of sites with PD ≥ 6 mm also improved significantly (*p* < 0.001) in both groups after three and six months; however, the reduction of PD was higher in the AMX/MET than in the MET group at three months (0.42 mm in median; *p* = 0.011) and at six months (0.32 mm in median; *p* = 0.032).

### 3.2. Tested Bacteria Associated with Periodontal Disease

The absence (no detection) of *A. actinomycetemcomitans* at baseline was a requirement for entering the clinical trial. Rechecking again after three and six months also did not detect that bacterial species. The counts of *P. gingivalis* and *T. forsythia* were significantly reduced in both groups after three and six months (each *p* < 0.01). At three months, *T. denticola* decreased only in the AMX/MET group (*p* < 0.01), at six months in both groups (each *p* < 0.05). At three months, the counts of T. forsythia were less in the AMX/MET group than in the MET group (*p* = 0.042), T. denticola by trend (*p* = 0.075). All other differences between the groups were not statistically significant. The results are given in Table 3.

### 3.3. Biomarkers

No statistically significant difference was found at intra- and inter-group testing. However, by trend, the levels of IL-1β and MMP-8 decreased to a higher degree in the AMX/MET than in the MET group after three months (*p* = 0.096 and 0.084, Table 4).

## 4. Discussion

Subgingival instrumentation significantly reduces probing depth, clinical attachment level and bleeding on probing in periodontitis patients [6,7,8]. The adjunctive therapy with systemic antibiotics in patients with severe affected sites by periodontitis improves these clinical results further and reduces the need for surgical periodontal therapy [11,30].

In the present study, two antibiotic regimens, a combination of two antibiotics and a single antibiotic administration were compared. In both groups, metronidazole was applied; in one group, amoxicillin was also applied. When using only a single antibiotic, side effects by the second might be avoided. A major side effect of amoxicillin is an allergic reaction; about 8% of the US population has a history of penicillin allergy [31]. The systematic review by Teughels et al. [11] suggests more adverse effects by AMX/MET than by other antibiotics in periodontal therapy.

Initially, the combination of amoxicillin and metronidazole was introduced to treat “*A. actinomycetemcomitans*-associated” periodontitis [32]. In vitro, it was shown that amoxicillin augments the uptake of metronidazole into the bacterial cell and induces in such a way a synergistic effect in killing the bacteria by the antibiotics [33]. Meanwhile, the combination of amoxicillin and metronidazole is the most used antibiotic regimen in periodontal therapy; its adjunctive benefit over subgingival instrumentation alone was justified in several systematic reviews [11,23,34].

Amoxicillin and metronidazole are administered in different dosages (250–500 mg amoxicillin and 200–500 mg metronidazole per application), in general applied three times per day, for different periods (from 3 days up to 14 days) [11,23,35]. In our study, the used dosage (500 mg amoxicillin/400 mg metronidazole) was given to the patients three times per day for seven days following the schedule of Harks 2015 [36].

In the present study, the PD reduction of sites with initial PD ≥ 6 mm (primary outcome) was significantly better in the AMX/MET than in the MET group. This was not accompanied by a significant higher reduction in the number of sites with PD ≥ 6 mm. When considering only the need for periodontal surgery (PD ≥ 6 mm requires further periodontal therapy), Matuliene et al.’s [37] administration of solely metronidazole might be sufficient. In the AMX/MET group, the reduction was a median of 2.5 mm after three months, which remained stable after six months. The reduction of PD and the attachment gain are in coincidence to improvements reported in the literature; higher than those reported by Boia et al. [38] and Morales et al. [39] but lower than in the study of Duarte et al. [40] or Mombelli et al. [41]. Our study reports improvements of sites with initial ≥6 mm as described by Duarte et al. [40] to evaluate the status of closed pocket and incomplete periodontal therapy related to the necessity of additional surgical periodontal therapy [37,42].

Clinical studies comparing metronidazole alone with amoxicillin and metronidazole in periodontal therapy are rare. One study with 66 participants in four study groups (inclusive solely amoxicillin and placebo groups) reported higher improvements of PD, CAL and BOP after six months when amoxicillin + metronidazole was adjunctively administered than metronidazole alone [43]. Another study with a follow-up of one year and also including a placebo group resulted in clinical superiority of both antibiotic regimens but just had a trend (no statistical significance) to the better outcome of the combined antibiotic therapy versus metronidazole alone [44]. To the best of the authors’ knowledge, after a thorough search of the literature, no paper was found that addressed the comparison between the adjunctive therapy with amoxicillin/metronidazole or metronidazole in periodontitis patients tested negative for *A. actinomycetemcomitans* in the subgingival biofilm before nonsurgical periodontal treatment.

The better results for amoxicillin and metronidazole than for metronidazole alone in absence of *A. actinomycetemcomitans* raise once more the question of the significance of microbiological diagnostics in the treatment planning of periodontal therapy. It might be the goal of an antibiotic therapy to target bacterial species playing a crucial role in the pathogenesis of the disease and to preserve the commensals [45]. For that the identification of potential pathogens might be of interest. Clinical results, also after adjunctive antibiotic therapy, often did not see any association with the detection of selected microorganisms, e.g., *A. actinomycetemcomitans* [41,46]. Otherwise, our retrospective analysis suggested that patients with *P. gingivalis*, *T. forsythia* and *T. denticola* benefit from adjunctive amoxicillin/metronidazole. On the other hand, it should be also seriously questioned if adjunctive antibiotics are really needed in periodontal therapy [24]. An analysis including more than 300 patients revealed that patients younger than 55 years and having more than 35 % of PD ≥ 5 mm benefit most from adjunctive amoxicillin and metronidazole [13]. Considering the increasing resistance to antibiotics, it is of interest to identify patients who will complete the periodontal therapy successfully only with adjunctive antibiotics.

The few available in vitro data on antibiotic resistance suggest a high susceptibility rate of *T. forsythia* to metronidazole [47,48]. However, in the present study, AMXTMET showed an even stronger depletion of *T. forsythia* and *T. denticola*. A synergistic effect of amoxicillin with metronidazole has to be assumed. A direct antibacterial synergism was not found against P. gingivalis, but neither T. forsythia nor T. denticola were tested [49]. Combinations of antibiotics augment their intracellular activities, as shown for moxifloxacin when combined with metronidazole in epithelial cells infected with *P. gingivalis* [50].

The combination of amoxicillin with metronidazole was also found to be more active than metronidazole alone in diverse biofilm models. The combination of the antibiotics decreased the metabolic activity in a three-species biofilm model of *Streptococcus sanguinis*, *Fusobacterium nucleatum* and *P. gingivalis*, and, in a ten-species biofilm model, the combined administration of antibiotics reduced the bacterial counts and strengthened *P. gingivalis* but not *T. forsythia* or *T. denticola* [51].

In the present study, periodontal inflammation seemed to be more reduced in the AMX/MET than in the MET group. This can be concluded from the lower results of BOP by trend (no statistical significance) after six months as well as from lower inflammatory biomarkers in the GCF, IL-1β and MMP-8, by trend (no statistical significance) after three months. It might be an indirect effect via changed microbiota or by a direct effect of antibiotics on the immune response; e.g., amoxicillin is able to decrease the release of the proinflammatory tumor-necrosis factor (TNF)-α from human monocytes exposed to lipopolysaccharides [52]. Metronidazole reduced the release of inflammatory cytokines from periodontal ligament fibroblasts stimulated with the LPS of *P. gingivalis* [53]. Wang et al. [54] found a decrease of IL-6 in the serum of rats after the adjunctive administration of amoxicillin and metronidazole during nonsurgical periodontal therapy. Al-Khureif et al. [55] reported increased levels of IL-10 and reduced levels of lL-17 in the gingival crevicular fluid of periodontitis patients after subgingival instrumentation and adjunctive antibiotic therapy with amoxicillin and metronidazole. These studies did not include groups with just one of these combined used antibiotics. Thus, it remains unclear if the nonbacterial effect is due to amoxillin or metronidazole.

There are some limitations of the present study. A third group without adjunctive antibiotic treatment as a negative control was not included. Moreover, the patients were not blinded to the antibiotics.

In summary, within the limits of the present study, it can be concluded that metronidazole is weaker than amoxicillin/metronidazole as an adjunct to subgingival instrumentation in patients with severe periodontitis and that tested *A. actinomycetemcomitans* negative in the subgingival biofilm. The adjunctive systemic use of amoxicillin/metronidazole results in better clinical and microbiological outcomes and is recommended in all patients who need adjunctive antibiotics in periodontal therapy.

## 5. Conclusions

When *A. actinomycetemcomitans* is not detected in the subgingival biofilm, the adjunctive systemic use of amoxicillin/metronidazole results in better clinical and microbiological outcomes than metronidazole alone of non-surgical periodontal therapy when, after a careful weighted decision considering the potential side effects, the application of systemic antibiotics is scheduled.

## Figures and Tables

**Table 1:** Demographic data in the MET (subgingival instrumentation+metronidazole) and the AMX/MET groups (subgingival instrumentation+metronidazole/amoxicillin) at baseline (before subgingival instrumentation).

Variable	MET Group (*n* = 29)	AMX/MET Group (*n* = 29)	*p*
Age (years; mean ± SD)	55.14 ± 11.48	52.17 ± 10.16	0.171
Male/female, n	20/9	18/11	0.581
Smoker, n	16	15	0.792

**Table 2:** Clinical variables (median, 25 percentile, 75 percentile) at baseline, after three and six months in the MET (subgingival instrumentation+metronidazole; *n* = 29) and the AMX/MET groups (subgingival instrumentation+metronidazole/amoxicillin; *n* = 29).

Variable	Baseline	3 Months	P (Baseline–3 Months)	6 Months	P (Baseline–6 Months)	Difference Baseline–3 Months	Difference Baseline–6 Months
Mean CAL (mm)							
MET	4.58 (4.21/5.01)	3.77 (3.59/4.03)	<0.001 *	3.77 (3.55/4.15)	<0.001 *	0.72 (0.41/1.20)	0.61 (0.45/1.11)
AMX/MET	4.67 (4.14/4.0)	3.73 (3.61/4.08)	<0.001 *	3.77 (3.64/4.21)	<0.001 *	0.84 (0.49/1.15)	0.65 (0.45/1.19)
P	0.988	0.988		0.630		0.852	0.822
BOP (%)							
MET	75.9 (37.64/100)	12.8 (10.6/15.9)	<0.001 *	13.1 (0.9/8.99)	<0.001 *	64.7 (19.6/87.4)	59.9 (19.9/86.7)
AMX/MET	81.0 (44.49/100)	13.7 (11.9/15.9)	<0.001 *	12.6 (11.7/19.2)	<0.001 *	56.1 (25.7/86.4)	53.3 (30.3/87.6)
P	0.955	0.262		0.785		0.780	0.697
Mean PD (mm)							
MET	4.44 (4.16/4.83)	3.64 (3.48/3.80)	<0.001 *	3.67 (3.50/3.79)	<0.001 *	0.69 (0.47/1.22)	0.66 (0.46/1.21)
AMX/MET	4.55 (4.15/4.95)	3.62 (3.47/3.91)	<0.001 *	3.69 (3.66/3.9)	<0.001 *	0.92 (0.5/1.1)	0.83 (0.47/1.18)
P	0.602	0.932		0.355		0.474	0.816
Number of sites with PD ≥ 6 mm							
MET	27 (16.5/44)	5 (2/8.5)	0.001 *	4 (2/9)	<0.001 *	20 (14/39)	21 (13.5/37)
AMX/MET	33 (17/43)	6 (2/8.5)	<0.001 *	5 (3.5/9.5)	<0.001 *	26 (15/37)	28 (14.5/38)
P	0.641	0.956		0.894		0.549	0.570
Mean PD of sites with initial PD ≥ 6 mm (mm)							
MET	7.04 (6.47/7.44)	4.95 (4.65/5.38)	<0.001	4.86 (4.45/5.31)	<0.001 *	2.09 (1.44/2.66)	2.19 (1.73/2.76)
AMX/MET	7.19 (6.67/7.61)	4.68 (4.30/5.01)	<0.001	4.68 (4.30/5.02)	<0.001 *	2.51 (2.00/2.76)	2.51 (2.12/2.88)
P	0.234	0.052	0.197			0.011 ^#^	0.032 ^#^

* statistically significant difference—Wilcoxon signed rank test within the group vs. baseline; ^#^ statistically significant difference—Mann–Whitney U test between the groups.

**Table 3:** Bacterial counts (log10, median, 25 percentile, 75 percentile)/sample of *Aggregatibacter actinomycetemcomitans*, *Porphyromonas gingivalis*, *Treponema denticola* and *Tannerella forsythia* at baseline, after three and six months in the MET (subgingival instrumentation+metronidazole) and the AMX/MET groups (subgingival instrumentation+metronidazole/amoxicillin).

	Baseline	3 Months	P (Baseline–3 Months)	6 Months	P (Baseline–6 Months)
*A. actinomycetemcomitans*					
MET	0 (0/0)	0 (0/0)	n.a.	0 (0/0)	n.a.
AMX/MET	0 (0/0)	0 (0/0)	n.a.	0 (0/0)	n.a.
P	n.a.	n.a.		n.a.	
*P. gingivalis*					
MET	4.36 (0/5.55)	0(0/3.37)	0.001 *	0 (0/3.24)	0.001 *
AMX/MET	4(0/5.83)	0 (0/0)	0.001 *	0 (0/2.12)	0.003 *
P	0.647	0.288		0.353	
*T. forsythia*					
MET	5.84 (5.5/6.4)	4.23 (3.77/5.4)	0.001 *	4.8 (3.24/5.55)	0.004 *
AMX/MET	5.76 (5.25/6.31)	3.56 (2.60/4.68)	<0.001 *	4.53 (3.73/5.17)	0.002 *
P	0.652	0.042 ^#^		0.613	
*T. denticola*					
MET	3.15 (0/4.32)	2.52 (0/3.56)	0.140	2.28 (0/3.44)	0.028 *
AMX/MET	3.54 (0/4.8)	0 (0/3.25)	0.005 *	2.78 (0/3.5)	0.027 *
P	0.358	0.075		0.416	

* statistically significant difference—Wilcoxon signed rank test within the group vs. baseline; ^#^ statistically significant difference—Mann–Whitney U test between the groups.

**Table 4:** Biomarker levels (pg/sample; median (25 percentile/75 percentile) at baseline, after three and six months in the MET (subgingival instrumentation+metronidazole) and the AMX/MET groups (subgingival instrumentation+metronidazole/amoxicillin).

	Baseline	3 Months	P (Baseline–3 Months)	6 Months	P (Baseline–6 Months)
MMP-8					
MET	1794.8 (805.7/2977.0)	758.52 (359.7/2224.0)	0.424	2358.08 (815.6/6478.1)	0.471
AMX/MET	1776.81 (702.5/4123.2)	722.64 (233.9/8593.0)	0.096	2132.96 (145.2/7099.4)	0.495
P	0.886	0.528		0.461	
IL-1β					
MET	18.24 (7.39/41.11)	8.51 (2.35/18.16)	0.949	17.62 (6.19/44.98)	0.471
AMX/MET	18.17 (11.83/35.71)	10 (2.92/29)	0.084	9.49 (3.12/41.89)	0.909
P	0.702	0.414		0.268	

Wilcoxon signed rank test: no significant longitudinal changes compared to baseline; Mann–Whitney U test: no significant differences between the groups.
